# Severe COVID-19 Illness: Risk Factors and Its Burden on Critical Care Resources

**DOI:** 10.3389/fmed.2020.583060

**Published:** 2020-11-19

**Authors:** Kyongsik Yun, Jeong Seok Lee, Eun Young Kim, Himanshu Chandra, Baek-Lok Oh, Jihoon Oh

**Affiliations:** ^1^Computation and Neural Systems, California Institute of Technology, Pasadena, CA, United States; ^2^GENOME INSIGHT Inc., Daejeon, South Korea; ^3^Division of Trauma and Surgical Critical Care, Department of Surgery, Seoul St. Mary's Hospital, College of Medicine, The Catholic University of Korea, Seoul, South Korea; ^4^Anderson School of Management, University of California, Los Angeles, Los Angeles, CA, United States; ^5^Department of Ophthalmology, Seoul National University Hospital, Seoul, South Korea; ^6^Department of Psychiatry, Seoul St. Mary's Hospital, College of Medicine, The Catholic University of Korea, Seoul, South Korea

**Keywords:** ECMO—extracorporeal membrane oxygenation, ventilator, severity, COVID-19, hydroxychloroquine

## Abstract

In South Korea, the first confirmed case of coronavirus 2019 (COVID-19) was detected on January 20, 2020. After a month, the number of confirmed cases surged, as community transmission occurred. The local hospitals experienced severe shortages in medical resources such as mechanical ventilators and extracorporeal membrane oxygenation (ECMO) equipment. With the medical claims data of 7,590 COVID-19 confirmed patients, this study examined how the demand for major medical resources and medications changed during the outbreak and subsequent stabilization period of COVID-19 in South Korea. We also aimed to investigate how the underlying diseases and demographic factors affect disease severity. Our findings revealed that the risk of being treated with a mechanical ventilator or ECMO (critical condition) was almost twice as high in men, and a previous history of hypertension, diabetes, and psychiatric diseases increased the risk for progressing to critical condition [Odds Ratio (95% CI), 1.60 (1.14–2.24); 1.55 (1.55–2.06); 1.73 (1.25–2.39), respectively]. Although chronic pulmonary disease did not significantly increase the risk for severity of the illness, patients with a Charlson comorbidity index score of ≥5 and those treated in an outbreak area had an increased risk of developing a critical condition [3.82 (3.82–8.15); 1.59 (1.20–2.09), respectively]. Our results may help clinicians predict the demand for medical resources during the spread of COVID-19 infection and identify patients who are likely to develop severe disease.

## Background

The first case of coronavirus 2019 (COVID-19) in South Korea was detected on January 20, 2020, and the cumulative number of confirmed cases was 31 by February 19. However, since a large outbreak occurred in Daegu on February 20, the number of cases increased drastically to >9,000 in 1 month (1). The health care system faced an unprecedented crisis during this period, with newly diagnosed COVID-19 patients increasing by more than 1,000 per day, which was compounded by a critical shortage in medical resources. As recent studies widely report on the risk stratification (2) and mortality rate in COVID-19 (3), we aimed to study the impact of the COVID-19 pandemic on the utilization of medical resources and to evaluate the potential risk factors associated with the severity of the illness.

## Methods

We used the database of South Korea's Health Insurance Review and Assessment Service (HIRA), which contains data for 7,590 COVID-19 patients (4). Based on the medical claims forms for patients treated for COVID-19, which outline their treatment and previous medical history, we classified disease severity into three categories (mild, severe, and critical) and identified potential risk factors for the severity of illnesses. The classification of disease severity was based on the guidelines of the Korea Center for Disease Control and Prevention (K-CDC). If a patient received oxygen therapy (by nasal cannula or mask), they were classified as a “severe” case. If a patient required a ventilator for breathing or if there was a claim history of Extra Corporeal Membrane Oxygenation (ECMO) use, they were classified as a “critical” case (5). The quantity and frequency of the use of medical resources were calculated from January 20 to May 15 (Supplementary Methods). The selection and classification process of participants is presented as a CONSORT flowchart (Supplementary Figure 1).

## Results

**Figure 1:**
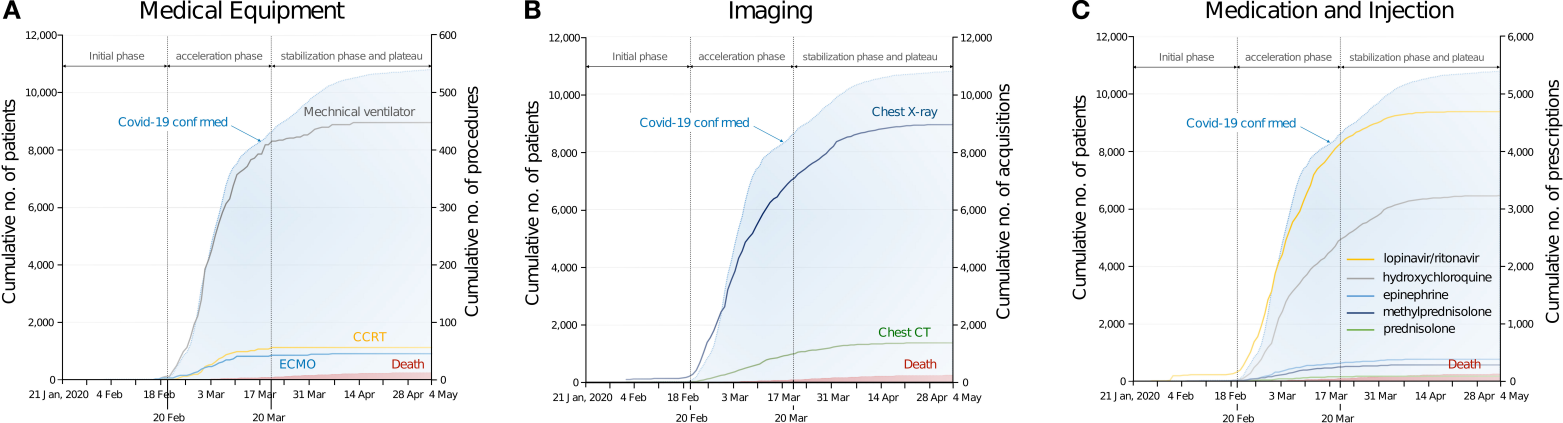
Utilization of medical resources during the spread of COVID-19. **(A)** Cumulative number of procedures of medical equipment (mechanical ventilator, extracorporeal membrane oxygenation, and continuous renal replacement therapy). **(B)** Cumulative number of medical imaging. **(C)** Cumulative number of medications. Number of COVID-19 confirmed patients and COVID-19 related deaths are based on the entire patients (*n* = 10,801, as of May 4, 2020), and graph of medical resources based on the dataset of the medical claims submitted to the Health Insurance Review and Assessment Service (*n* = 7,590).

In the initial phase (when the number of daily confirmed cases was below 100, in the period up to February 19), the demands for mechanical ventilators were 0.3 per day, which increased to ~13.7 per day during the acceleration phase (from when the number of daily confirmed cases exceeded 100 and reached 80% of the total number of COVID-19 diagnosis; from February 20 to March 20); this then decreased to 0.7 per day during the stabilization and plateau phase (from the end of acceleration phase to May 15; Figure 1). Extracorporeal membrane oxygenation (ECMO) comprised 80% of the total requests on March 9 during the acceleration phase. The demand for continuous renal replacement therapy (CRRT) comprised 80% of claims on March 7, 2 days earlier than that for ECMO.

The number of imaging claims such as for chest computed tomography and chest X-ray reached 80% of the total claims after the acceleration phase (March 24 and March 21, respectively). Among the COVID-19 related medications, the use of lopinavir/ritonavir increased rapidly following the outbreak, and claims for it reached 80% of the total number of prescriptions during the acceleration phase (March 14). Claims for hydroxychloroquine reached 80% of the saturation point during the stabilization phase (March 23). In South Korea, the total number of claims for lopinavir/ritonavir was the highest (*n* = 4,696) compared to that for other medications.

Patients with confirmed COVID-19 were also classified into three (mild, severe, and critical) groups and we analyzed each group based on their demographic and medical characteristics (Tables 1, 2 and Supplementary Methods). Mortality rates were seen to increase with age, and among patients >80 years, 36% of men and 25% of women died from COVID-19. More than 8% of men over the age of 70 years were mechanically ventilated while 1.8% of men aged 60–69 years required ECMO. The use of ventilators and CRRT equipment was less common at all ages for women than for men.

**Table 1:** Demographic characteristics and use of medical equipment in COVID-19 patients by age groups.

**Outcome**	**Men (*N* = 3,095)**	**Women (*N* = 4,495)**
Primary outcome: mortality	121/3,095	106/4,495
Death within 7 in-hospital days—no./total no. (%)	43/3,095 (1.4)	39/4,495 (0.9)
Death after 7 in-hospital days—no./total no. (%)	78/3,095 (2.5)	67/4,495 (1.5)
Primary outcome: mortality by age groups—no./total no. (%)		
−19	0/226 (0)	0/205 (0)
20–39	1/1,188 (0.1)	1/1,441 (0.1)
40–49	1/331 (0.3)	0/675 (0)
50–59	10/526 (1.9)	4/975 (0.4)
60–69	26/444 (5.9)	10/615 (1.6)
70–79	39/258 (15.1)	28/333 (8.4)
>80	44/122 (36.1)	63/251 (25.1)
Secondary outcomes: in-hospital days by age groups [mean (SD)]		
0–19	18.0 (9.9)	16.4 (8.6)
20–39	17.8 (9.5)	16.4 (9.2)
40–49	17.9 (11.3)	17.8 (10.5)
50–59	17.6 (11.3)	19.9 (11.2)
60–69	20.4 (11.6)	21.4 (12.3)
70–79	19.9 (12.7)	20.8 (12.2)
>80	19.1 (12.2)	21.4 (13.9)
Secondary outcomes: usage of mechanical ventilator by age groups—no./total no. (%)		
0–19	0/226 (0)	0/205 (0)
20–39	3/1,188 (0.3)	0/1,441 (0)
40–49	1/331 (0.3)	0/675 (0)
50–59	8/526 (1.5)	10/975 (1.0)
60–69	30/444 (6.8)	13/615 (2.1)
70–79	23/258 (8.9)	17/333 (5.1)
>80	10/122 (8.2)	15/251 (6.0)
Secondary outcomes: usage of CRRT by age groups—no./total no. (%)		
0–19	0/226 (0)	0/205 (0)
20–39	0/1,188 (0)	0/1,441 (0)
40–49	2/331 (0.6)	0/675 (0)
50–59	2/526 (0.4)	3/975 (0.3)
60–69	7/444 (1.6)	1/615 (0.2)
70–79	3/258 (1.2)	3/333 (0.9)
>80	0/122 (0)	5/251 (2.0)
Secondary outcomes: usage of ECMO by age groups—no./total no. (%)		
0–19	0/226 (0)	0/205 (0)
20–39	0/1,188 (0)	0/1,441 (0)
40–49	1/331 (0.3)	0/675 (0)
50–59	1/526 (0.2)	3/975 (0.3)
60–69	8/444 (1.8)	1/615 (0.2)
70–79	0/258 (0)	3/333 (0.9)
>80	1/122 (0.8)	5/251 (2.0)

**Table 2:** Characteristics of COVID-19 patients according to disease severity^a^.

**Subgroup**	**Severity of illnesses**		
	**Mild^**b**^ (*N* = 6,636)**	**Severe^**c**^ (*N* = 680)**	**Critical^**d**^ (*N* = 47)**
Sex^*^			
Men	2,637 (39.7)	311 (45.7)	26 (55.3)
Women	3,999 (60.3)	369 (54.3)	21 (44.7)
Admission type^***^			
Transfer from other hospital	85 (1.3)	25 (3.7)	5 (10.6)
Emergency medical service	302 (4.6)	9 (1.3)	1 (2.1)
Others	5,828 (87.8)	644 (94.7)	41 (87.2)
Region of inpatient care^***^			
Daegu (outbreak area)	2,298 (34.6)	389 (57.2)	20 (42.6)
Others	4,338 (65.4)	291 (42.8)	27 (57.4)
**Current illnesses**^e^^***^			
Hypertension	1,352 (20.4)	381 (56.0)	30 (63.8)
Diabetes	1,210 (18.2)	314 (46.2)	23 (48.9)
Chronic pulmonary diseases	1,601 (24.1)	205 (30.1)	15 (31.9)
Cerebrovascular disease	469 (7.1)	155 (22.8)	9 (19.1)
Psychiatric diseases	2,033 (30.6)	379 (55.7)	24 (51.1)
Types of national health insurance^*^			
Health insurance	6,126 (92.3)	605 (89.0)	44 (93.6)
Medical care	510 (7.7)	75 (11.0)	3 (6.4)

a *Deaths from COVID-19 disease were not included*.

b *Confirmed patient who had never been administered neither mechanical ventilator nor ECMO*.

c *Confirmed patients who had been administered oxygen treatment*.

d *Confirmed patients who had been administered mechnical ventilator or ECMO*.

e *Duplicate diagnosis were allowed*.

*Numbers in parentheses denote percentages in each severity category*.

* *Chi-square analysis, p <0.05*.

*** *Chi-square analysis, p <0.001*.

Of the 7,363 patients who survived at the time of the analysis, 6,636 developed mild illness (no oxygen therapy or mechanical ventilators needed) whereas 680 developed severe conditions and were managed with oxygen therapy. A further 47 developed critical conditions, requiring mechanical ventilators or ECMO. Among the mild and severe cases, the incidence rate of COVID-19 was higher in women than in men (mild: 40 men, 69 women; moderate: 46 men, 54 women), and vice versa in critical cases (55 men, 45 women). The severity of COVID-19 also differed depending on the region where the inpatient care was administered (regardless of the area of incidence) and the patients' pre-existing medical conditions (Table 2).

It was found that men were more likely to have a fatal outcome or die than women [odds ratio for women (95% CI), 0.43 (0.33–0.57); Table 3], and patients with hypertension, diabetes, and psychiatric illnesses were also at higher risk than those without these pre-existing conditions [hypertension = 1.60 (1.14–2.24), diabetes = 1.55 (1.55–2.06), psychiatric diseases = 1.73 (1.25–2.39)]. Patients with a weighted Charlson Comorbidity Index (CCI) of 3–4 showed 2.8 times greater risk (95% CI: 2.76–5.97) of developing critical illness than patients with a CCI of 0, whereas a CCI exceeding 5 increased the risk by 3.8 times (3.82–8.15). Patients who were treated in the Daegu area, which experienced extremely high community transmission, had a 1.58-fold increased risk (1.20–2.09) of developing critical illness than those treated in other regions.

**Table 3:** Risk of severity in COVID-19 patients.

	**Odds ratio^**a**^**	**95% CI**	***P*-value**
**Sex**^**b**^			
Men	1 (ref)		
Women	0.43	0.33–0.57	<0.001
**Age**			
20–39	0.14	0.05–0.41	<0.001
40–49	0.08	0.01–0.58	0.01
50–59	1(ref)		
60–69	2.50	1.53–4.11	<0.001
70–79	5.41	5.41–8.86	<0.001
>80	15.87	9.62–26.19	<0.001
**Current illnesses**^**b**^			
Hypertension	1.60	1.14–2.24	<0.05
Diabetes	1.55	1.55–2.06	<0.05
Chronic pulmonarydiseases	1.13	0.85–1.50	0.39
Cerebrovascular disease	0.87	0.64–1.18	0.37
Psychiatric diseases	1.73	1.25–2.39	<0.05
**Charson comorbidity index**^**c**^			
Weighted CCI 0	1 (ref)		
Weighted CCI 1–2	1.89	0.87–4.09	0.11
Weighted CCI 3–4	2.76	2.76–5.97	<0.05
Weighted CCI >=5	3.82	3.82–8.15	<0.05
**Region of inpatient care**^**b**^			
Daegu (outbreak area)	1.58	1.20–2.09	<0.05

a *Risk of critical condition or death relative to mild or severe condition*.

b *Adjusted for sex, age, hypertension, diabetes, pulmonary diseases, cerebrovascular diseases, psychiatric diseases, and region of inpatient care*.

c *Adjusted for sex, age, Charson comorbidity index, and region of inpatient care*.

## Discussion

During the COVID-19 pandemic in South Korea, the risk of receiving ECMO or mechanical ventilation in COVID-19 infection was approximately two times higher in men, and the risk increased by more than 1.5 times when patients suffered from chronic diseases such as hypertension or diabetes. A previous history of respiratory or cerebrovascular diseases did not affect the risk of developing critical conditions or causing death. These findings are consistent with recent reports that demonstrated the number of comorbidities (6), hypertension, and diabetes (7) to be associated with an increased risk for disease severity.

Although South Korea faced a shortage of intensive care unit (ICU) facilities after the outbreak in Daegu city (51 of 54 negative pressurized beds were occupied within 4 days after the first confirmed diagnosis in this region) (8), there were no disruptions in critical care treatment systems. This is because the number of ICU beds per 100,000 people is relatively high (28.5 per 100,000 population) among Organization for Economic Co-operation and Development (OECD) countries. Moreover, the government implemented measures to secure critical care units immediately after the COVID-19 outbreak (9).

Recent statistics show that the infection rate among healthcare workers in South Korea is relatively lower (0.4%) (8) than that in other countries, particularly the USA (Seattle) where 5.3% cases were recorded in front-line workers (10). This difference in the number of infections is likely attributable to a high rate (90.1%) of mild cases (~6,636 of 7,363 cases) of COVID-19 infections and hospitals implementing a stricter screening procedure at the initial stage of the outbreak (8).

This study had several limitations that need to be acknowledged. First, the established risk factors known to contribute toward the prognosis of COVID-19 infection, such as the current respiratory function and usage of immunosuppressants, were not investigated in this study. Given that the dataset of HIRA contained data on medical claims only, it was not possible to analyze the laboratory and biochemical measures for assessing the risk factors for disease severity. Second, each patient was classified into mutually exclusive groups of mild, severe, and critical; therefore, it was impossible to discern the recovery from severe or critical conditions and the worsening of disease severity. However, despite these limitations, our results might help clinicians identify high-risk groups for COVID-19 infection and predict medical resource requirements during the ongoing COVID-19 outbreak.

## Data Availability Statement

The datasets generated in this study can be found in online repositories. The names of the repository/repositories and accession number(s) can be found below: https://hira-covid19.net/.

## Ethics Statement

The studies involving human participants were reviewed and approved by The Catholic University of Korea (KC20ZISI0259). Written informed consent for participation was not required for this study in accordance with the national legislation and the institutional requirements.

## Author Contributions

KY, B-LO, and JO conceived of the presented idea. KY, HC, and B-LO performed and analyzed the dataset. JO, JL, and HC verified the analytical methods. B-LO and JO supervised the findings of this work. All authors discussed the results and contributed to the final manuscript.

## Conflict of Interest

JL was employed by GENOME INSIGHT Inc. The remaining authors declare that the research was conducted in the absence of any commercial or financial relationships that could be construed as a potential conflict of interest.

## References

[1] The Updates on COVID-19 in Korea Available online at: https://www.cdc.go.kr/board/board.es?mid=a30402000000&bid=0030 (accessed April 30, 2020).

[2] Knight SR Ho A Pius R Buchan I Carson G Drake TM. Risk stratification of patients admitted to hospital with covid-19 using the ISARIC WHO clinical characterisation protocol: development and validation of the 4C mortality score. BMJ. (2020) 370:m3339. 10.1136/bmj.m3339 32907855 PMC7116472

[3] Mehra MR Desai SS Kuy S Henry TD Patel AN Cardiovascular disease, drug therapy, and mortality in covid-19. N Engl J Med. (2020) 382:e102 10.1056/NEJMoa2007621 32356626 PMC7206931

[4] #opendata4COVID19 Available online at: https://COVID19data.hira.or.kr. (accessed June 11, 2020).

[5] Korea Centers for Disease Control and Prevention Guideline for Covid-19 Infection (Ver 7.0). Korea Centers for Disease Control and Prevention (2020).

[6] Liang W Liang H Ou L Chen B Chen A Li C. Development and validation of a clinical risk score to predict the occurrence of critical illness in hospitalized patients with COVID-19. JAMA Intern Med. (2020) 180:1–9. 10.1001/jamainternmed.2020.2033 32396163 PMC7218676

[7] Wu Z McGoogan JM. Characteristics of and important lessons from the coronavirus disease 2019 (COVID-19) outbreak in China: summary of a report of 72 314 cases from the Chinese Center for Disease Control and Prevention. JAMA. (2020) 323:1239–42. 10.1001/jama.2020.2648 32091533

[8] Kim JH An JAR Min PK Bitton A Gawande AA. How South Korea responded to the COVID-19 outbreak in Daegu. NEJM Catal Innov Care Delivery. (2020) 1:1–14. 32301298

[9] Ministry of Health and Welfare Nationally Designated Negative Pressure Isolation Rooms in Hospitals. Available online at: http://www.mohw.go.kr/react/al/sal0301vw.jsp?PAR_MENU_ID=04&MENU_ID=0403&page=1&CONT_SEQ=354783 (accessed October 3, 2020).

[10] Mani NS Budak JZ Lan KF Bryson-Cahn C Zelikoff A Barker GEC. Prevalence of coronavirus disease 2019 infection and outcomes among symptomatic healthcare workers in Seattle, Washington. Clin Infect Dis. (2020) ciaa761. 10.1093/cid/ciaa761 32548613 PMC7337651

