# Supplementary material for: Severe COVID-19 Illness: Risk Factors and Its Burden on Critical Care Resources
Source: Front Med (Lausanne). 2020 Nov 19;7:583060. doi: 10.3389/fmed.2020.583060 (PMC7711126; doi:10.3389/fmed.2020.583060)
Supplement: Supplementary file 1 [file Data_Sheet_1.docx]

**Supplementary Appendix**

This appendix has been provided by the authors to give readers additional information about their work.

**Severe COVID-19 illness: Risk Factors and its Burden on Critical Care Resources.**

Kyongsik Yun, Ph.D.,^1*^ Jeong Seok Lee, M.D.,^2*^ Eun Young Kim, M.D., Ph.D.,^3^ Himanshu Chandra, M.S.,^4^ Baek-Lok Oh, M.D.,^5†^ and Jihoon Oh, M.D., Ph.D.^6†^

**Table of Contents**

Supplementary Methods

**Supplementary Methods**

**Study Population and Definition**

The government of the South Korea shared the Covid-19 nationwide patient data to public through the website (https://hira-Covid19.net). This dataset is based on the medical claims to the Health Insurance Review and Assessment Service (HIRA) and comprised of 62 categories including demographics, dosage and types of prescribed medication, types of medical equipment used and information of health care providers.

As of June 13, 2020, this dataset consisted of medical claims of 234,427 persons, 476,508 cases that is related to Covid-19 infection. Claims data submitted to HIRA by May 15, 2020 were included and 7,590 patients who had confirmed Covid-19 infection by real-time PCR (polymerase chain reaction), and these patients were targeted in the analysis.

Classification of disease severity followed the guideline of Korea Center for Disease Control and Prevention (K-CDC); regardless of patients' age, one who had received oxygen therapy (by nasal cannula or mask) treated as a 'severe'. If he/she were breathing with the help of a ventilator and if there was a claim history of Extra Corporeal Membrane Oxygenation (ECMO) use, they were classified as 'critical' condition.^1^ Patients who died from Covid-19 infection were separately identified in the dataset.

As the HIRA's dataset is based on the date of medical claims in each hospital and clinic, the results presented in this study may differ from the actual administration date of medical equipment and medications.

**Variables**

Among 62 categories in the datasets, the code named 'GNL_CD' which designate the type of medical resources was firstly classified. Fifteen classes of medication (antiviral drugs, antibiotics, sympathomimetic drugs and vasoconstrictors), 3 types of radiological examination (Chest X-ray [AP, PA], Chest CT) that are widely used in the treatment of Covid-19 patients were extracted. Usage and a total number of treatment of oxygen therapy (code:M0040), ventilator (code: M58XX), continuous renal replacement therapy (CRRT; code: O7031) and ECMO (code: O190X) were also extracted.

According to the guideline of The Korean Society of Infectious Diseases, one or both of following two drugs were prescribed to Covid-19 patients depending on the disease severity; Kaletra (lopinavir and ritonavir) and hydroxyquinolone.^2^ Thus, we evaluated how much dosage of these drugs were used in Covid-19 patients depending on their severity of illnesses.

As about 78% of Covid-19 infection occurred in Daegu and its Province area (Gyeongbuk), the region where they had a treatment (code: RVD_PLC_CD) was evaluated in each patient. Then, we examined whether Covid-19 patients had a history of medical treatment with chronic illnesses such as hypertension (ICD codes: I00 to I15), diabetes (E10.0, E10.6, E10.8, E11.0, E11.6, E11.8, E12.0, E12.1, E12.6, E12.8, E12.9, E13.0, E13.6, E13.8, E14.0, E14.6, E14.8 E10.5, E10.7, E11.5, E11.7, E12.2-E12.5, E12.7, E13.5, E13.7, E14.5, E14.7), chronic pulmonary diseases (I27.8, I27.9, J40.x, J67.x, J68.4, J70.1, J70.3), cardiovascular disease (G45.x, G46.x, H34.0, I60.x-I68.x) and psychiatric diseases (F00 to F99) within 5 years. Type of national health insurance (code: INSUP_TP_CD) was also evaluated to indirectly outline the economic status of patients.

**Statistical Analysis**

All analyses were performed using R software (version 3.6.2).^3^ Descriptive characteristics were presented as number or frequency (%). The chi‐square test was used to determine the differences between categorical variables. To investigate the risk of critical condition or death, odd ratios and 95% confidential intervals were calculated using multivariable logistic regression in adjusted models. Model 1 was adjusted for age, sex, region of inpatient care, and status of chronic co-morbidity (hypertension, diabetes, chronic pulmonary disease, cerebrovascular disease, and psychiatric disease). Model 2 was adjusted for age, sex, region of inpatient care, and weighted Charlson comorbidity index (CCI). The cutoff *P* value of <0.05 was considered for statistical significance.

**References**

1. Korea Centers for Disease Control and Prevention. Guideline for Covid-19 Infection (ver 7.0). 2020;

2. The Korean Society of Infectious Diseases, Korean Society for Antimicrobial Therapy, The Korean Society of Pediatric Infectious Diseases. Guideline on the pharmaceutical treatment on Covid-19 infection. (In Korean). 2020;

3. R Core Team. A language and environment for statistical computing. R Foundation for Statistical Computing. Vienna, Austria, 2020;Available from: http://www.R-project.org/
