# Supplementary figures and images for: Severe COVID-19 Illness: Risk Factors and Its Burden on Critical Care Resources
Source: Front Med (Lausanne). 2020 Nov 19;7:583060. doi: 10.3389/fmed.2020.583060 (PMC7711126; doi:10.3389/fmed.2020.583060)

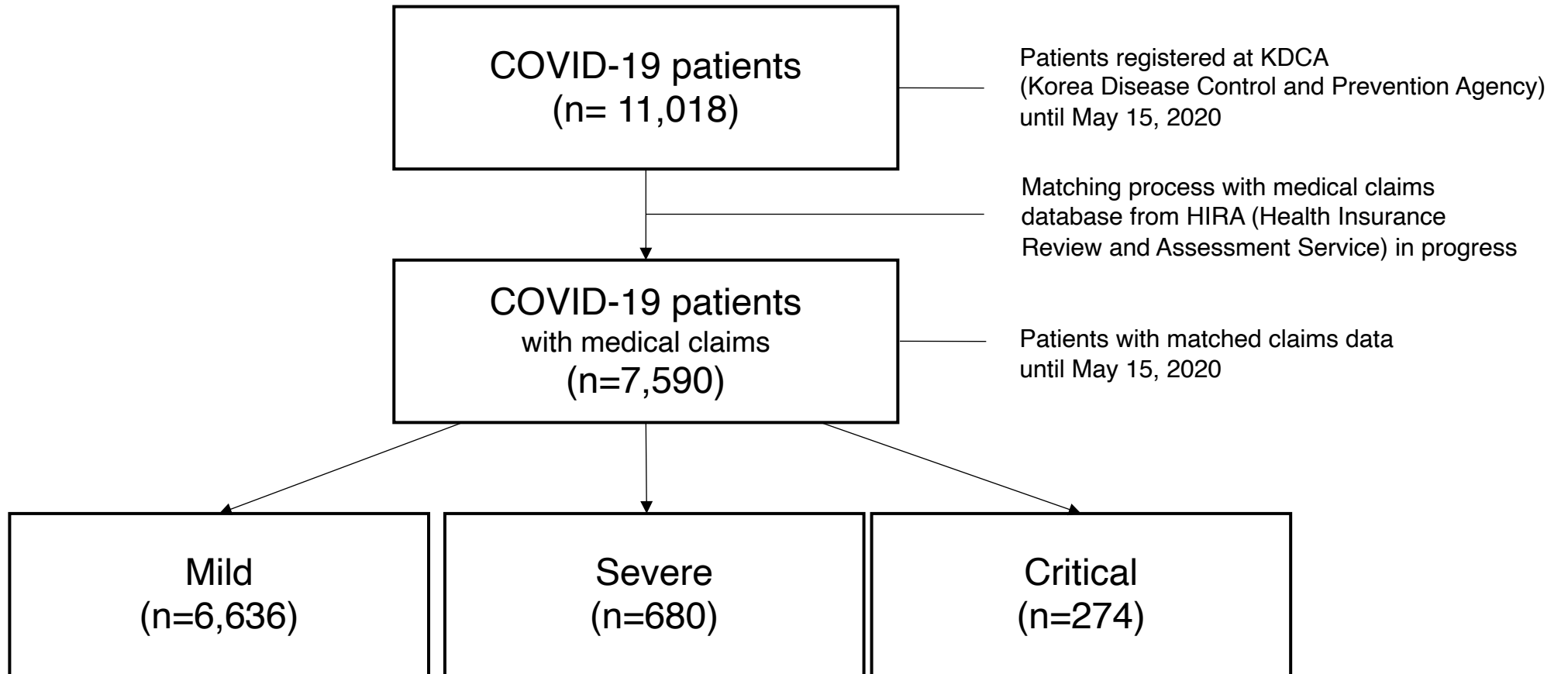

Supplement: Supplementary file 2 [file Image_1.pdf]
